# Comprehensive analysis of HIV reservoirs in elite controllers

**DOI:** 10.1172/JCI165446

**Published:** 2023-02-01

**Authors:** Brooke D. Kennedy, Jana Blazkova, Jesse S. Justement, Victoria Shi, M. Ali Rai, Maegan R. Manning, Lauren Praiss, Kathleen Gittens, Paul A. Wender, Sean Patro, Xiaolin Wu, Susan Moir, Tae-Wook Chun

**Affiliations:** 1 Laboratory of Immunoregulation, National Institute of Allergy and Infectious Diseases, and; 2 Clinical Center, NIH, Bethesda, Maryland, USA.; 3 Department of Chemistry, Stanford University, Stanford, California, USA.; 4 Frederick National Laboratory for Cancer Research, Frederick, Maryland, USA.

**Keywords:** AIDS/HIV, Virology, Molecular biology, T cells

**To the Editor:** HIV remains an uncurable disease in infected individuals receiving antiretroviral therapy (ART). Despite unparalleled research efforts to find a cure, there is a growing sentiment in the field that complete viral eradication is unlikely with existing therapies. Therefore, an approach involving immune-mediated virologic control without ART represents a realistic goal, even if the HIV reservoir persists (1). In this regard, the ability of some individuals to control HIV replication (i.e., elite controllers [ECs]) illustrates the feasibility of achieving ART-free virologic remission. Prior studies have established that ECs carry low HIV DNA burdens and exhibit robust HIV-specific immune responses (2). However, there are few studies directly comparing the composition and frequencies of HIV reservoirs in ECs with those in chronically infected individuals receiving ART (CAs). We conducted the present study to address this issue.

We studied 9 ECs and 22 CAs (Supplemental Table 1 and Supplemental Methods; supplemental material available online with this article; https://doi.org/10.1172/JCI165446DS1). Blood products were obtained in accordance with a protocol approved by the Institutional Review Board of the National Institute of Allergy and Infectious Diseases of the NIH. All participants provided written informed consent. We first compared the composition and size of HIV reservoirs in the EC and CA groups. The levels of total HIV DNA (*P* < 0.0001), cell-associated HIV RNA (*P* < 0.0001), intact proviral DNA (IPD) (*P* = 0.0026), and defective HIV DNA (*P* < 0.0001) in CD4^+^ T cells were significantly lower in the EC group compared with those in the CA group (Figure 1, A and B) (2). To assess the replication competency of infected cells, we measured the levels of inducible virion-associated HIV RNA (ivRNA) and replication-competent virus in CD4^+^ T cells. In contrast to the above data, there was no significant difference in the levels of ivRNA (*P* = 0.4187) and replication-competent HIV (*P* = 0.9303) between the two groups (Figure 1C). Three ECs had exceptionally low infectious HIV burdens in their CD4^+^ T cells. EC-6 and EC-7 had no detectable infectious virus (<1 copy in 245 × 10^6^ CD4^+^ T cells), despite repeated testing. The ratio of intact-to-defective HIV DNA was similar in the EC and CA groups (*P* = 0.7471; Figure 1D); however, the ratio of IPD to replication-competent virus was significantly lower in the EC group compared with that in the CA group (*P* = 0.0052; Figure 1D).

The frequencies of IFN-γ^+^ and CD107a^+^IFN-γ^+^TNF-α^+^MIP-1β^+^ HIV Gag-specific CD8^+^ T cells were significantly higher in the EC group compared with those in the CA group (*P* = 0.0315 and *P* = 0.0001, respectively; Figure 1E), suggesting that ECs may express sufficient viral antigens.

IPD burdens were relatively high in EC-4 and EC-6 (75 and 105 copies/10^6^ cells, respectively). Therefore, we conducted longitudinal measurements of ivRNA and IPD (EC-4) and near full-length sequencing of HIV DNA from a sorted CD4^+^ T subset (EC-6). Despite aviremia, the levels of ivRNA and IPD increased over time in EC-4 (Figure 1F), potentially signaling the eventual loss of virologic control. In EC-6, the majority of IPD was found in the effector memory (Tem) CD4^+^ T cell subset (Figure 1G). Using near-full-length single-genome amplification, we obtained 88 sequences from the Tem CD4^+^ T cell subset of EC-6. Although all clones had intact HIV env, 90% displayed a gag start codon mutation and a deletion in the pol gene (designated as group 1), 8% had a deletion at the *gag* start site (group 2), and 2% had a deletion in the *gag/pol* genes (group 3, see Supplemental Figure 1). All sequences had deletions in the major splice donor site and the dimerization initiation sequence (Supplemental Figure 1). These data demonstrated that the 79 clones (group 1) identified as intact by the IPD assay (IPDA) were replication defective, further explaining undetectable infectious HIV in EC-6.

Potential explanations for the discordant findings between the PCR- and cell culture–based assays include the possibility that not all IPD are replication competent. Although the IPDA offers an in-depth look at reservoirs at the molecular level (3), a previous study suggested that the IPDA also detects replication-defective viruses (4). It is plausible that a higher ratio of IPD to replication-competent HIV in CAs versus ECs is due to detection of defective DNA by the IPDA.

Indeed, the sequencing analysis of IPD in EC-6 was shown to be replication defective. Yet this does not explain why ECs still maintain infectious HIV reservoirs in vivo. A study analyzing HIV DNA integration sites showed that the genomic landscape of IPD in ECs may prevent viral expression, thereby contributing to virologic control (5). However, we showed that infectious virus can be recovered from ECs, with the exception of those with exceptionally low or replication-defective DNA. Additionally, evidence for ongoing HIV replication has been demonstrated in certain ECs (6).

Major caveats of our work include the small number of ECs examined and the lack of integrated provirus analyses. Nonetheless, we demonstrated that the inclusion of cell culture–based assays measuring infectious viral burdens and whole-genome sequencing would be necessary to accurately assess HIV reservoirs in certain infected populations. Additionally, our data suggest that the presence of replication-competent HIV does not preclude ART-free virologic suppression and frequent monitoring of the viral reservoir size in ECs may identify potential virologic failure.

## Supplementary Material

Supplemental data

## Figures and Tables

**Figure 1:**
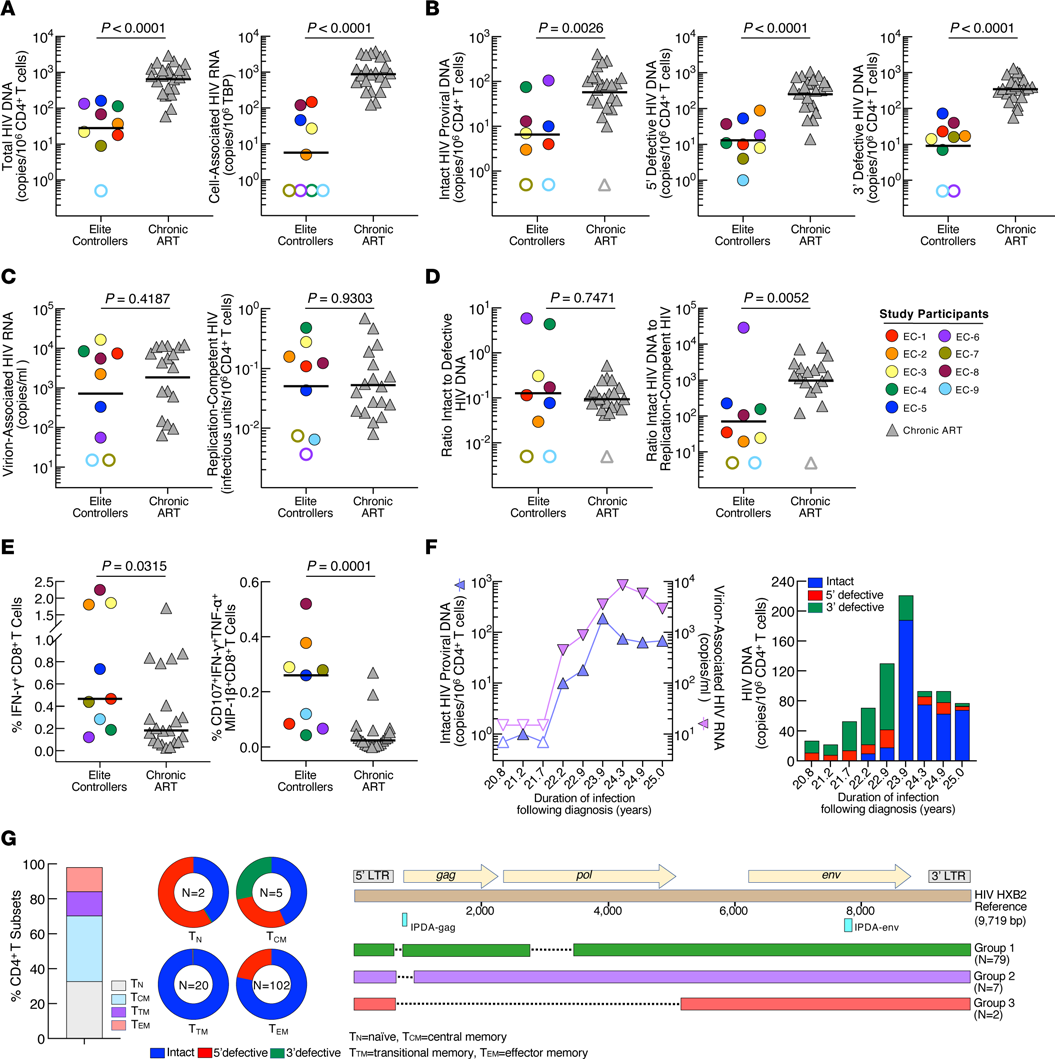
HIV reservoirs and immune responses. (**A**) Total HIV DNA and cell-associated HIV RNA, TBP, TATA-binding protein, (**B**) intact proviral and defective DNA, (**C**) virion-associated HIV RNA and replication-competent virus, (**D**) ratios of intact-to-defective and intact proviral DNA to replication-competent virus, and (**E**) HIV-specific immune responses between the EC and CA groups. Open symbols indicate values under limits of detection. Black bars represent the geometric mean or median values. *P* values were determined using the Mann-Whitney test and were not adjusted. (**F**) Measurements of intact proviral DNA and inducible virion-associated HIV RNA as well as composition of intact and defective HIV DNA in EC-4. (**G**) Percentage of CD4^+^ T cell subsets and distribution of intact and defective HIV DNA within each subset in EC-6. *n* = total DNA copy numbers/10^6^ CD4^+^ T cells. Sequence alignment of HIV DNA is shown.

## Version 1 02/01/2023

Electronic publication
