# Supplementary material for: Comprehensive analysis of HIV reservoirs in elite controllers
Source: J Clin Invest. 2023 Feb 1;133(3):e165446. doi: 10.1172/JCI165446 (PMC9888367; doi:10.1172/JCI165446)
Supplement: Supplemental data [file jci-133-165446-s202.pdf]

**Supplemental Materials**

## Supplemental Methods

### Study participants

Our study consisted of 9 elite controllers (ECs) and 22 HIV-infected individuals who initiated ART during the chronic phase of infection (termed Chronic ART [CAs], Supplemental Table 1) and who participated in a previous study (1). All ECs were diagnosed with HIV for more than 6 months, ART-naïve, had ≥90% of plasma viremia measurements below the limits of detection, maintained $CD4^+$ T cell counts >450 cells/$mm^3$, and had no AIDS defining illnesses at the time of study. The median duration of HIV infection was 24.3 years (range 4.8-35) and 14 years (range 5.2-32.8) for the EC and CA groups, respectively ($P$=0.3968). For the ART-treated group, the median duration of treatment was 7.7 years (range 2.5-19.8).

### Measurements of HIV reservoirs

Levels of total HIV DNA in $CD4^+$ T cells were measured by droplet digital PCR (Bio-Rad Laboratories). Genomic DNA was isolated from purified $CD4^+$ T cells (QIAGEN) and digested (MscI; New England BioLabs) prior to PCR amplification with HIV-specific and RPP30-specific primers and probes. For amplification of the HIV LTR, the following primers and probes were used: 5’-GRAACCCACTGCTTAAGCCTCAA -3’ (5’ primer), 5’-TGTTCGGGCGCCACTGCTAGAGA -3’ (3’ primer), and 5’-6FAM-AGTAGTGTGTGCCCGTCTGTT-IABkFQ-3’ (probe). For amplification of RPP30, the following primers and probes were used: 5’-GATTTGGACCTGCGAGCG-3’ (5’ primer), 5’-

GCGGCTGTCTCCACAAGT-3’ (3’ primer), and 5’-HEX-TTCTGACCTGAAGGCTCTGCGC-IABkFQ-3’ (probe). HIV DNA copy numbers were normalized per $1x10^6$ $CD4^+$ T cells.

Levels of cell-associated HIV RNA were measured by reverse transcriptase PCR (RT-PCR) and ddPCR (Bio-Rad Laboratories) from purified $CD4^+$ T cells. The RNeasy Mini Kit (QIAGEN) was used to isolate RNA for cDNA synthesis with qScript XLT cDNA Master Mix (Quanta Biosciences) according to the manufacturer’s instructions. cDNA product was quantified using HIV-specific and TATA box-binding protein (TBP)-specific primers and probes. For HIV RNA (unspliced), the following primers and probes were used: 5’-TCTCTAGCAGTGGCGCCCGAACA-3’ (5’ primer), 5’- TCTCCTTCTAGCCTCCGCTAGTC-3’ (3’ primer), 5’-6FAM- CAAGCCGAGTCCTGCGTCGAGAG-IABkFQ-3’ (probe). For TBP, the following primers and probes were used: 5′-CACGAACCACGGCACTGATT-3′ (5’ primer), 5′-TTTTCTTGCTGCCAGTCTGGAC-3′ (3’ primer), and 5′-HEX-TGTGCACAGGAGCCAAGAGTGAAGA-IABkFQ-3′ (probe). Copy numbers of HIV RNA were normalized per $1x10^6$ copies of the housekeeping gene TBP.

Levels of intact and defective HIV DNA were measured by the intact proviral DNA assay (IPDA) using digital PCR (QIAGEN) and modified primers and probes. Genomic DNA was isolated with the QIAamp DNA Mini Kit (QIAGEN) from purified $CD4^+$ T cells and amplified with HIV-specific and RPP30-specific primers and probes. For HIV Gag, the following primers and probes were used: 5’-GACTAGCGGAGGCTAGAAGGAGAGA-3’ (5’ primer), 5’-CTAATTCTCCCCCGCTTAATAYTGACG-3’ (3’ primer), 5’-6FAM-A+T+GGG+TG+CGAGA-IABkFQ-3’ (LNA probe). For HIV Env, the following primers and probes were used: 5’- AGTGGTGCAGAGAGAAAAAAGAGC-3’ (5’ primer), 5’-GTCTGGCCTGTACCGTCAGC-3’ (3’ primer), 5’-VIC- CCTTGGGTTCTTGGGA-MGB-3’

(probe) and 5'- CCTTAGGTTCTTAGGAGC-MGB-3' (unlabeled hypermutated probe). For housekeeping gene RPP30, the following primers and probes were used: 5'-GATTTGGACCTGCGAGCG-3' (RPP30-1 5' primer), 5'- GCGGCTGTCTCCACAAGT-3' (RPP30-1 3' primer), 5'-6FAM-TTCTGACCTGAAGGCTCTGCGC-IABkFQ-3' (RPP30-1 probe), 5'- GTGTGAGTCAATCACTAGACAGAA-3' (RPP30-2 5' primer), 5'-AAACTGCAACAACATCATAGAGC-3' (RPP30-2 3' primer), and 5'-HEX-AGAGAGCAACTTCTTCAAGGGCCC-IABkFQ-3' (RPP30-2 probe). For the elite controller study group, the HIV-specific reactions were performed in 8 technical replicates, and the RPP30-specific reactions were performed in 4 technical replicates. For the ART-treated study group, both HIV and RPP30-specific reactions were performed in 3 technical replicates. Copy numbers were normalized per $1x10^6$ $CD4^+$ T cells and intact HIV DNA copies were adjusted with the DNA shearing index (DSI).

Levels of inducible HIV RNA were measured by the inducible HIV assay. Replicates of $1x10^7$ $CD4^+$ T cells were stimulated in 2 ml of media containing interleukin-2 (IL-2) and a phorbol ester compound (SW13, 50nM) for 48 hours at 37°C. Following incubation, supernatant was collected separately for each replicate and centrifuged to remove any residual cells. Virion-associated HIV RNA was quantified in 1 ml of cell-free supernatant by the COBAS AmpliPrep/COBAS TaqMan HIV-1 Test, version 2.0 (COBAS). Average of replicates was reported as the level of virion-associated HIV RNA per sample.

Level of $CD4^+$ T cells carrying replication-competent HIV was determined by quantitative co-culture assay using serially diluted (1,000,000, 200,000, 40,000, 8,000, 1,600, and 320 cells per well) and replicates of $5x10^6$ $CD4^+$ T cells from study participant. Each well was incubated with anti-CD3 antibodies and irradiated PBMCs from healthy HIV-negative

donors. After 1 day incubation, $1x10^6$ CD8-depleted and anti-CD3-stimulated PBMCs from HIV-negative donors were added to each well, followed by removal of cell suspensions and replenishment with fresh medium containing IL-2 every 3 days. HIV p24 ELISA was performed on the culture supernatants to identify wells containing replication-competent HIV.

**Fluorescent activated $CD4^+$ T cell sorting**

Cryopreserved PBMCs were thawed and enriched for $CD4^+$ T cells (StemCell Technologies). Cells were stained with the following fluorophore-conjugated surface-marker antibodies: CD4-PerCP (clone SK3), CD45RA-FITC (clone HI100), CD27-APC (clone M-T271), and CCR7-PE (clone 3D12) and sorted through BD FACS Aria Fusion flow cytometer (BD Biosciences) with BD FACS Diva software version 9.0. $CD4^+$ T cells were sorted based on surface-marker expression: $CD27^+CD45RA^+CCR7^+$ (naïve), $CD27^+CD45RA^-CCR7^+$ (central memory), $CD27^+CD45RA^-CCR7^-$ (transitional memory), and $CD27^-$ (effector memory).

**Measurements of HIV-specific immune response**

Frequencies of HIV Gag-specific $CD8^+$ T cells were determined by intracellular cytokine staining. PBMCs were incubated for 6 hours at 37°C with overlapping HIV-1 Gag peptides (HIV Reagent Program), brefeldin A (Sigma-Aldrich), and anti-CD107a-BV510 antibody. Cells were stained with the following fluorophore-conjugated antibodies for surface markers: CD3-BUV805 (clone SK7), CD4-BUV395 (clone M-T477), and CD8-BUV737 (clone SK1). Stained cells were fixed and permeabilized prior to incubation with the following intracellular antibodies IFN-γ-APC (clone B27), TNF-α-BV650 (clone MAb11) and MIP-1ß-PE (clone D21-1351). Data were collected using the SpectroFlo software (Cytek Biosciences) version 2.20 on the Cytek Aurora cytometer and analysis was performed using FlowJo version 10.7.1.

**Near-full-length (NFL) sequencing of HIV DNA**

HIV DNA was titrated by limiting dilution to ensure single copy amplification per reaction. All PCRs were performed using SuperFI DNA polymerase (2U/µl) (ThermoFisher) and the following cycling parameters: 98°C for 3 mins; 98°C for 10 sec, 60°C for 30 sec, 72°C for 5 min (repeat 30x); 72°C for 5 min. First round PCR was performed with NFL1 primers: 5'-AGTCAGTGTGGAAAATCTCT*A*G-3' (5' primer) and 5'-GAGGGATCTCTAGTTACCAG*A*G-3' (3' primer). First round product was diluted with 80µl of 5 mM Tris-HCl. Second round PCR was performed with NFL2 primers: 5'-BBBBBBBBBBGTGGAAAATCTCTAGCAGT*G*G-3' (5' primer) and 5'-BBBBBBBBBBTTACCAGAGTCACACAACAG*A*C-3' (3' primer). Asterisks indicate phosphorothioated DNA bonds to limit 3' to 5' exonuclease activity. BBBBBBBBBB indicates 10bp barcode sequences. Barcodes were incorporated during second round PCR and NFL products were identified by running second round products on 1% agarose gel with 1 kb plus DNA ladder (ThermoFisher) or using QIAxcel (QIAGEN) per the manufacturers' instructions. Barcoded products were pooled and cleaned with AMPure XP (Beckman Coulter) according to the manufacturer's instructions.

Sequencing was performed using PacBio Sequel lle HIFI and Pacbio Amplicon-Seq standard protocol (Pacific Biosciences). Over 3 million circular consensus reads (CCS) with a base call quality of QV score 43.1 were generated from a single flow cell. CCS reads were demultiplexed with barcodes using a custom Perl script. A minimum of 100 CCS reads were interrogated to generate consensus sequences for each amplicon. Approximately 20 of the 100 CCS reads were identical to the consensus sequence for any given amplicon while the rest of CCS reads often had a random 1bp difference from the consensus sequence (8.2-8.7 kb) with

>99.9% identity. The consensus sequences for all 88 amplicons were then used for sequence alignment using the HIVAlign tool to the HIV HBX reference genome (https://www.hiv.lanl.gov/content/sequence/VIRALIGN/viralign.html). Among the 88 amplicons, three groups of sequences were identified: Group 1 was the most abundant (N=77) followed by Group 2 (N=7), and Group 3 (N=2) (Genbank accession no. OP820703-OP820790). The NFL amplicon did not have full LTR sequence but the alignment in Figure 1G assumed no major LTR defect.

**Supplemental Table 1. Clinical characteristics of study participants.**

| | **Elite Controllers** (n=9) | **Chronic ART** (n=22) |
|---|---|---|
| **Sex** | | |
| Male (%) | 6 (66.7) | 20 (90.9) |
| Female (%) | 3 (33.3) | 2 (9.1) |
| **Age* (years)** | | |
| Median (interquartile range) | 54 (38-65) | 51 (45-56) |
| Range | 33-71 | 38-61 |
| **Race** | | |
| African American (%) | 2 (22.2) | 9 (40.9) |
| Caucasian (%) | 7 (77.8) | 10 (45.5) |
| Multiple Race (%) | 0 (0.0) | 1 (4.5) |
| Unknown (%) | 0 (0.0) | 2 (9.1) |
| **T cell count*** (cells/mm$^3$) | | |
| Median CD4$^+$ (interquartile range) | 778 (593-824) | 767 (591-964) |
| Range | 491-1,046 | 466-1,778 |
| Median CD8$^+$ (interquartile range) | 572 (355-824) | 626 (419-1,017) |
| Range | 296-1,400 | 296-1,587 |
| **Duration of infection following diagnosis*** (years) | | |
| Median (interquartile range) | 24.3 (7.0-32.1) | 14.0 (10.1-18.8) |
| Range | 4.8-35.0 | 5.2-32.8 |
| **Duration of ART*** (years) | | |
| Median (interquartile range) | N/A | 7.9 (3.4-11.4) |
| Range | N/A | 2.5-19.8 |
| **HLA Types** | | |
| HLA B27 (%) | 3 (33.3) | 0 (0.0) |
| HLA B57 (%) | 4 (44.4) | 2 (9.1) |

*At time of study

**Supplemental Figure 1: HIV proviral DNA sequence annotation and intactness.**

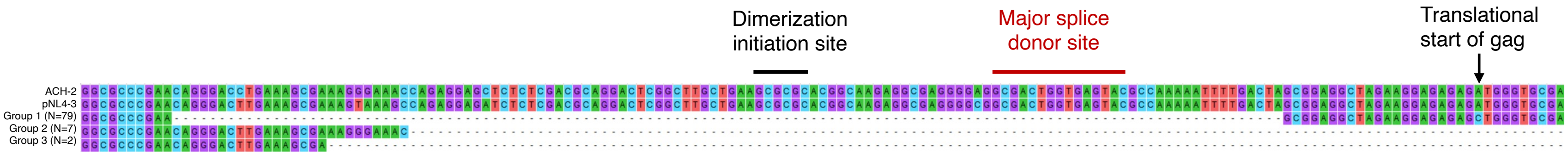


| ID | Seq length | Inferred intactness | Sequence completeness | 5' LTR length | TAR stem loop insertion | TAR stem loop deletion | PolyA insertion | PolyA deletion | U5/gag pair R1 insertion | U5/gag pair R1 deletion | Half PBS insertion | Half PBS deletion | MSD status | Package insertion | Package deletion | U5/gag pair R2 insertion | U5/gag pair R2 deletion | 2 base before gag status | gag start codon | gag insertion | gag deletion |
|---|---|---|---|---|---|---|---|---|---|---|---|---|---|---|---|---|---|---|---|---|---|
| ACH2 | 9730 | Intact | Complete | 634 | 0 | 0 | 0 | 0 | 0 | 0 | 0 | 0 | correct | 0 | 0 | 0 | 0 | correct | correct | 0 | 0 |
| pNL4-3 | 9709 | Intact | Complete | 634 | 0 | 0 | 0 | 0 | 0 | 0 | 0 | 0 | correct | 0 | 0 | 0 | 0 | correct | correct | 0 | 0 |
| Group 1 (N=79) | 8242 | Defective | gagstart_mut/pol_del | 17 | 0 | 61 | 0 | 6 | 0 | 11 | 0 | 0 | missing | 0 | 73 | 0 | 0 | correct | mutation | 0 | 0 |
| Group 2 (N=7) | 8756 | Defective | gagstart_del/gag_del | 17 | 0 | 61 | 0 | 6 | 0 | 11 | 0 | 0 | missing | 0 | 116 | 0 | 11 | deletion | deletion | 0 | 160 |
| Group 3 (N=2) | 4551 | Defecttive | gagstart_del/gag+pol_del | 17 | 0 | 61 | 0 | 6 | 0 | 11 | 0 | 0 | missing | 0 | 116 | 0 | 11 | deletion | deletion | 0 | 1503 |

The Proviral Sequence Annotation & Intactness Test (ProSeq-IT; psd.cancer.gov/tools/pvs_annot.php) tool was used to assess genome intactness of the three observed proviral sequences (2) compared to intact proviral sequence from ACH-2 cell line and pNL4-3 (GenBank MN691959.1, M19921.2). Deletion(s) in the 5' region (Figure 1G) render all proviruses defective due to major splice donor/packaging deletions, *gag* start mutation/deletion, and significant gag, gag/pol coding deletions. The 5'LTR was not included in amplification and sequencing. Sequences are displayed using MEGA7.

**Acknowledgements**

We thank the volunteers for their participation in this study. This work was supported by the Intramural Research Program of the NIAID, NIH and NCI contract 75N91019D00024.

**Author contributions**

BDK and TWC designed the research. BDK, JB, JSJ, VS, MAR, MRM, SP, XW, and TWC performed the research. LP, KG, and PAW contributed research materials. BDK, JB, SP, XW, SM, and TWC analyzed the data. BDK, SM, and TWC wrote the manuscript. All authors reviewed and approved the manuscript.

**Supplemental references**

1. Sneller MC, Huiting ED, Clarridge KE, Seamon C, Blazkova J, Justement JS, et al. Kinetics of Plasma HIV Rebound in the Era of Modern Antiretroviral Therapy. *J Infect Dis.* 2020;222(10):1655-9.
2. Shao W, Shan J, Hu WS, Halvas EK, Mellors JW, Coffin JM, et al. HIV Proviral Sequence Database: A New Public Database for Near Full-Length HIV Proviral Sequences and Their Meta-Analyses. *AIDS Res Hum Retroviruses.* 2020;36(1):1-3.
